# Altered heat nociception in cockroach *Periplaneta americana* L. exposed to capsaicin

**DOI:** 10.1371/journal.pone.0194109

**Published:** 2018-03-08

**Authors:** Justyna Maliszewska, Sonia Marcinkowska, Anna Nowakowska, Hanna Kletkiewicz, Justyna Rogalska

**Affiliations:** Department of Animal Physiology, Faculty of Biology and Environmental Protection, Nicolaus Copernicus University, Toruń, Poland; St. Joseph's Hospital and Medical Center, UNITED STATES

## Abstract

Some natural alkaloids, e.g. capsaicin and camphor, are known to induce a desensitization state, causing insensitivity to pain or noxious temperatures in mammals by acting on TRP receptors. Our research, for the first time, demonstrated that a phenomenon of pharmacological blockade of heat sensitivity may operate in American cockroach, *Periplaneta americana* (L.). We studied the escape reaction time from 50°C for American cockroaches exposed to multiple doses of different drugs affecting thermo-TRP. Capsaicin, capsazepine, and camphor induced significant changes in time spent at noxious ambient temperatures. Moreover, we showed that behavioral thermoregulation in normal temperature ranges (10–40°C) is altered in treated cockroaches, which displayed a preference for warmer regions compared to non-treated insects. We also measured the levels of malondialdehyde (MDA) and catalase activity to exclude the secondary effects of the drugs on these processes. Our results demonstrated that increase in time spent at 50°C (five versus one trial at a heat plate) induced oxidative stress, but only in control and vehicle-treated groups. In capsaicin, capsazepine, menthol, camphor and AITC-treated cockroaches the number of exposures to heat had no effect on the levels of MDA. Additionally, none of the tested compounds affected catalase activity. Our results demonstrate suppression of the heat sensitivity by repeated capsazepine, camphor and capsaicin administration in the American cockroach.

## Introduction

Capsaicin from chili pepper is known to alter animals’ thermoregulatory processes. In 1970, Jancsó-Gábor et al. [1] demonstrated that capsaicin injection produces hypothermia in rats and guinea pigs. Large doses of this alkaloid induce a desensitization state in which animals become insensitive to the hypothermic action of capsaicin. Capsaicin-desensitized rats and guinea pigs became incapable of protecting themselves against overheating and responded with hyperthermia to high ambient temperatures (32–40°C) [1].

Later it was demonstrated that thermoregulatory changes induced by capsaicin were mediated by the TRPV1 receptor, long before this channel received its current name [2–3]. TRPV1 is a polymodal sensor of chemical and heat stimuli, which can be activated by increasing ambient temperature higher than 42°C [4–5]. However, temperatures of approximately 37°C can potentiate TRPV1 responses to capsaicin and other chemical agonists, including allicin constituent of garlic [6], piperine from black pepper [7], camphor [8] and resiniferatoxin [9], as well as endogenously occurring lipid molecules such as anandamide–an amide derivative of arachidonic acid [10]. Moreover, activation temperature of TRPV1 depends on the animal taxa and may be different from 42°C, as 38°C in *Xenopus tropicalis* frogs [11] or >25°C in zebrafish [12].

Exposure of TRPV1 channels to capsaicin not only causes their activation, but also induces the release of proinflammatory peptides, such as substance P or calcitonin gene-related peptide, and the generation of impulses transmitted via central fibers to the spinal cord where they are perceived as pain. A state of high-dose capsaicin-induced neuronal insensitivity to stimuli, which would normally activate TRPV-expressing neurons, is termed desensitization [13]. After desensitization a depletion of substance P is observed, which contributes to the long-lasting effect of capsaicin [14–15]. Additionally, camphor application results in acute desensitization of TRPV1, to a stronger extent than capsaicin treatment [8]. In the central nervous system of American cockroach *Periplaneta americana* we can find neurons containing a substance P-like peptide [16].

Nociceptive responses to heat were studied in *Drosophila* and showed the involvement of TRP channels, as well as amnesiac and straightjacket genes in thermal nociception [17–19] Xu et al. [17] used heat plate assay (45°C) to determine the role of Painless receptor in thermal nociception in adult flies. Painless, a TRPA subfamily member, was first discovered to participate in insects thermosensation. *Drosophila* larvae deprived of Painless also demonstrated diminished reactions to high ambient temperatures (42°C) [20]. Apart from TRP receptors, some other components may control nociceptive behavior in *Drosophila*. Behavioral responses to noxious heat (>40°C) are mediated by the amnesiac gene, which is predicted to encode a neuropeptide precursor involved in associative and non-associative learning [18]. Straightjacket α2δ3, a voltage-gated calcium channel subunit, is required for heat nociception in both larva and adult *Drosophila*. Similar to the fly, mice lacking of α2δ3 display impaired acute heat responses [19]. The structures involved in cockroach response to noxious heat are not known. To date, the roles for only two TRP channels in the American cockroach have been determined—TRPγ (TRPC subfamily) [21] and more recently TRPL [22].

In the present study, the thermal response of *Periplaneta americana* after repeated exposure to compounds known to affect thermo-TRP channels was evaluated. The purpose was to determine whether a phenomenon similar to capsaicin desensitization in mammals is observed in cockroaches (whole-animal desensitization state). As an indicator, the latency to escape from noxious temperatures was measured. Escape reaction time from a heat box set to high ambient temperatures was previously used as a bioassay for studying the analgesic effect of opiates in crickets of the genus *Pteronemobius*. It has been shown that the injection with morphine increases the escape reaction time from 54°C [23]. In the present study, six substances known to affect thermo-TRP channels were evaluated: capsaicin (1); capsazepine (2); menthol (3); thymol (4); camphor (5) and allyl-isothiocyanate (6). (1) Capsaicin is a TRPV1 activator that induces a desensitized state in mammals [1]. (2) Capsazepine is a competitive antagonist of capsaicin. Capsazepine administration via drinking water led to systemic desensitization in mice. During ten days of continuous oral administration of capsazepine increased paw-withdrawal latency to radiant heat stimulation was observed [24]. The sustained desensitization was not an effect of sensory excitation antagonism by capsaicin, but was due to activation of TRPA1 receptor. (3) Menthol can affect the activation of various TRP channels. In vitro it inhibited the insect cold receptor TRPL [25], while in bees menthol blocked HsTRPA (warmth receptor) and induced preference for warmer regions in thermal gradient [26]. Menthol is also known mammalian TRPM8 activator that induces warmth-seeking behavior and hyperthermia in rats [27–28]. (4) Thymol’s effect on insect thermoregulatory processes is unknown. In mammals thymol exerts no effect on body temperature [28], but in vitro research has shown, that it can inhibit insect cold receptor TRPL [25]. (5) Camphor activates insect cold receptor TRPL and inhibits Painless, which responds to noxious heat as described above [25;29]. Camphor has also been reported to activate HsTRPA and is proposed to activate warmth receptor dTRPA1 [26]. (6) Allyl-isothiocyanate (AITC) is known to activate two warmth receptors in insects in vitro–dTRPA1 and Painless [26;30]. Recently, it was demonstrated that *Helicoverpa armigera* TRPA1 (HarmTRPA1) is activated by AITC and also functions as a warmth sensor [31]. There is no data on the effect of AITC on insect thermal preferences, however activation of the mammalian TRPA1 by AITC induced thermogenesis and reduced heat diffusion in rats [28]. Camphor and capsaicin were shown to induce oxidative stress [32–33], therefore we decided to determine some aspects of oxidative state in the examined cockroaches to exclude the possibility that their response might be a result of secondary effects of drug action on oxidative stress.

## Material and methods

### Insects

Experiments were performed on adult American cockroaches, *Periplaneta americana* (L.), both sexes. Insects were reared at 26±2°C under a 12 hr:12 hr light-dark photoperiod regime in plastic containers. They were fed oat flakes and apples and provided with water *ad libitum*.

### Substances

The test compounds (Sigma Aldrich) were dissolved in ethyl alcohol and diluted to obtain the desired concentrations: capsaicin 0.0001mM and 0.1mM, capsazepine 0.0001mM, menthol 2mM, thymol 1mM, camphor 15mM, and AITC 3mM. Optimal drug concentrations for use in the following experiments were determined based on preliminary results and literature data. The control group was treated with water. To exclude the effect of solvent, one group of cockroaches was exposed to ethyl alcohol in the same concentration as used to the test drug solution (1%; vehicle group). The tested substances (10μl) were applied under the wings, on the mesothorax.

### Experimental design

#### Heat plate

**Fig 1:**
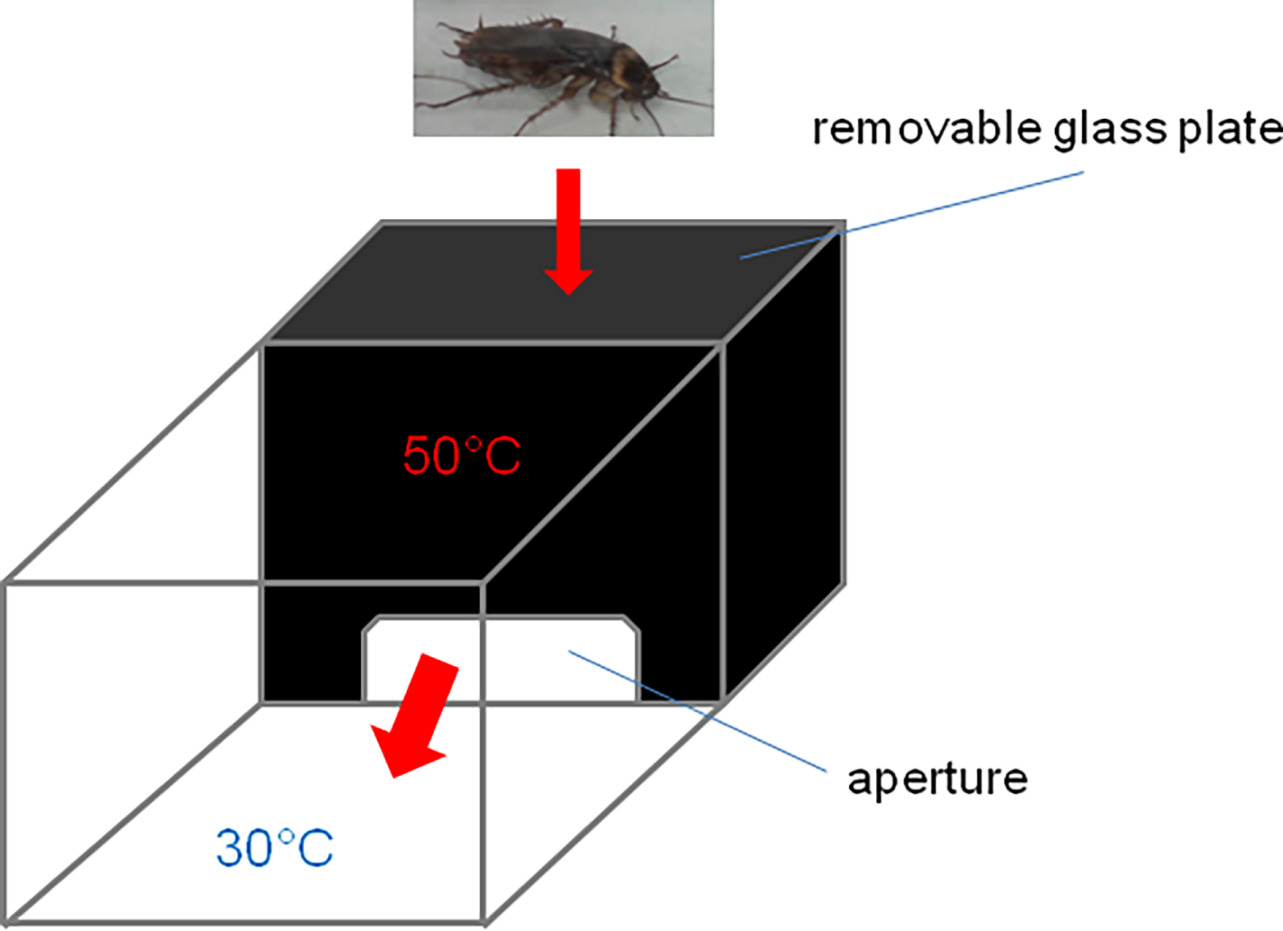
Heat box. Heat box used to measure the latency to escape from noxious temperature (50°C) in cockroaches.

To evaluate insect escape times from noxious heat, we constructed a ‘heat box’ (5×7×5 cm) (Fig 1). The temperature inside the chamber was set to 50°C and monitored by a UT 321 thermocouple. One side of the chamber had an aperture to allow the insect to escape. The insects were introduced to the box after removal of a dark glass plate on the upper surface. The interior of the heat chamber was dark in order to clearly distinguish the two areas: heat/dark area and cool/light area. Cockroaches exhibit negative phototaxis [34–35], therefore moving toward cool temperatures is associated with overcoming aversion to light. An individual cockroach was introduced to the hot compartment immediately after drug administration and the escape time was measured with a chronometer. The escape time measurement was immediately stopped once the insect exited through the aperture. Insects that did not exit the heat box after three minutes were excluded from further experiments (single cases as shown in S1 Spreadsheet).

**Fig 2:**
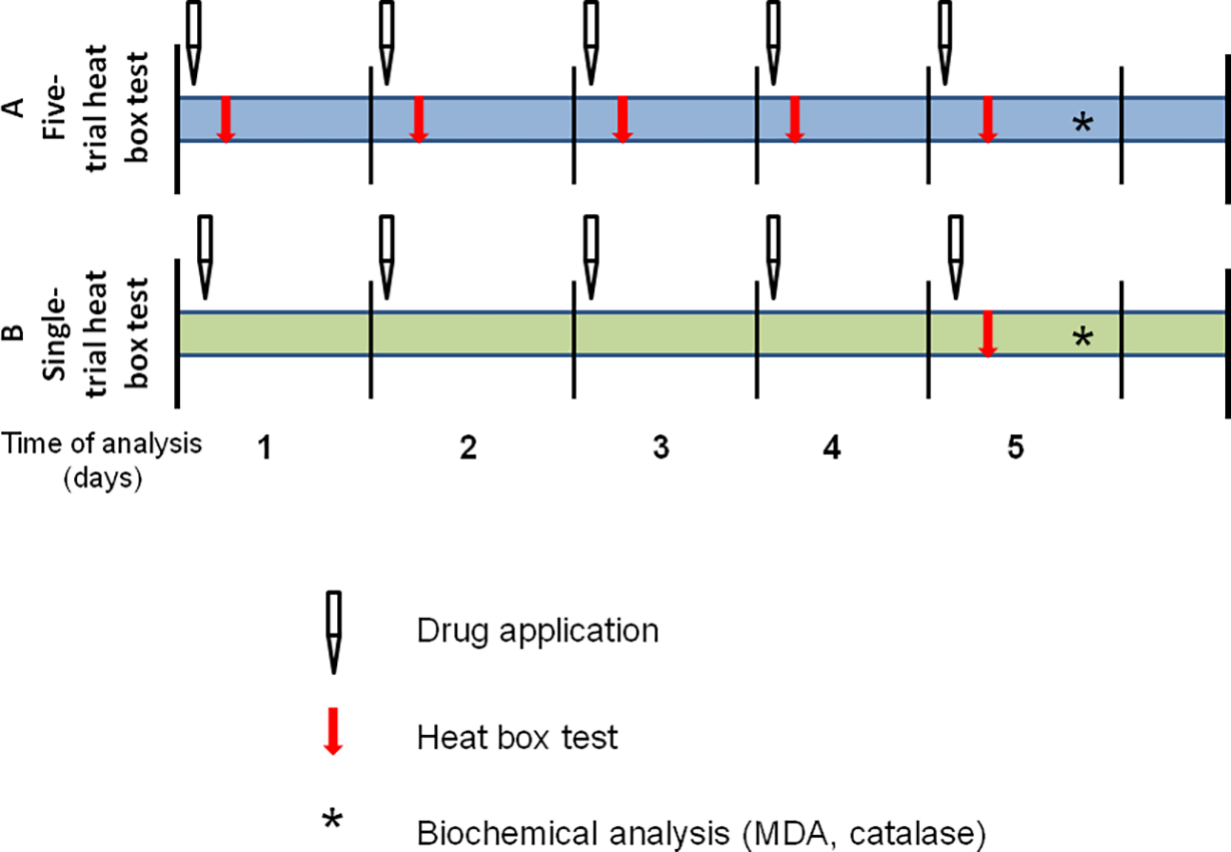
Experimental design. The scheme of the experimental design.

Two different experimental designs were used (Fig 2):

(A) Five-trial heat box test. Cockroaches were examined for five days (7 different drug groups; n = 13–60 for each treatment group). Each day they were applied with test drugs and tested in the heat-box (1 drug per subject/1 heat-box trial per day).
(B) Single-trial heat box test. The second part of experiment was performed to exclude effects of heat stress and learning on cockroach responses to agonists and antagonists of thermo-TRP. The test drugs were administered to the cockroaches once daily for five days, but the testing in the heat box was performed only after administration of the fifth dose on day five (one time in the heat box) (n = 16–39 for each treatment group; 1 drug per subject/ single trial on day five).

#### Behavioral thermoregulation

We have found that some TRP agonists resulted in increased time spent at high ambient temperatures (heat-box test), thus we decided to determine whether ‘sensitized’ insects alter their thermal preferences in the normal temperature range (10–40°C). A novel set of cockroaches was used in the behavioral thermoregulation experiment.

Experiments were performed in a thermal gradient using an identical method as described previously [36]. Briefly, after five days of drug administration, each individual cockroach (n = 12 for each drug) was placed in the thermal gradient trough and its thermal preferences were observed for 24 hours. The temperatures preferred by the insects were estimated from their positions in the thermal gradient.

### Oxidative stress

The test compounds affected cockroach responses to noxious high temperature. In order to assess whether the response might be a result of secondary effects of drug action on oxidative stress, lipid peroxidation by TBARS assay and catalase activity was measured.

#### Lipid peroxidation

The TBARS assay quantifies oxidative stress by measuring the level of lipid peroxidation. To assess the lipid peroxidation level, spectrophotometric quantitation of the thiobarbituric reaction product, malondialdehyde (MDA), was used [37–38]. Cockroaches (whole-body) were homogenized in cold phosphate buffer, followed by the addition of thiobarbituric acid solution (0.37% in 0.25M HCl), trichloroacetic acid solution (15% in 0.25M HCl), and butylated hydroxytoluene (BHT). The mixture was placed for 20 minutes in a boiling water bath, then cooled and transferred to Eppendorf tubes and centrifuged for 15 minutes (10 000×g; 25°C). Supernatant absorbance was measured at 532 nm. MDA concentration was determined based on the millimolar absorption coefficient (156 mmol^-1^ · L · cm^-1^).

#### Catalase activity

Catalase activity (CAT) was measured as described by Orta-Zavalza et al. [39]. Catalase activity was estimated as the decomposition rate of hydrogen peroxide. Briefly, cockroach whole-body homogenates were centrifuged and diluted 1:50 with 50mM phosphate buffer (pH 7.0). The diluted sample was mixed with phosphate buffer and 1ml of 10mM H_2_O_2_. The breakdown of H_2_O_2_ was immediately measured at 240 nm for 3 min, at 1 min intervals. The molar extinction coefficient for H_2_O_2_ at 240 nm is 34.9M^-1^cm^-1^. CAT activity was presented as unit/mg protein. One unit of CAT activity was defined as the amount that decomposes 1μmol H_2_O_2_/min at 25°C [40].

#### Protein concentration

The concentration of protein in the cockroach homogenates was determined by the Bradford method [41]. Bovine serum albumin (BSA) was used to construct the calibration curve.

### Data analysis

Kolmogorov-Smirnov test showed that the obtained data are not normally distributed, therefore the results were analyzed using non-parametric tests. The Mann-Whitney test showed that the there were no significant differences in escape time between males and females, so the subsequent analyses were performed on data obtained for both sexes. All analyses were performed with IBM SPSS Statistics 24 software. Data are presented as mean±SEM.

#### Latency to escape from heat plate

The effect of test compounds on latency to escape from heat plate was assessed using the Kruskal–Wallis test. If Kruskal–Wallis testing revealed a significant difference, a pairwise Mann-Whitney U test was carried out. Values were considered significant after adjustment of multiple testing with Holm’s sequential correction for multiple comparisons.

The differences between effects of separate doses of each tested drug in five-trial heat-box test (escape time of each dose was compared against each previous dose) were determined with Wilcoxon signed-rank test followed by Holm’s sequential correction for multiple comparisons.

#### Cockroach thermal preferences

The comparison between the effect of treatments on thermal preferences (means from 24 hour observation) was made using Kruskal–Wallis test followed by pairwise Mann-Whitney U test with Holm’s sequential correction for multiple comparisons.

#### Oxidative stress

The effect of the test drugs on MDA level and catalase activity was assessed with pairwise Mann-Whitney U tests after Kruskal–Wallis tests. Multiple testing adjustment after Mann-Whitney U tests were made with Holm’s sequential correction for multiple comparisons.

## Results

### Latency to escape from noxious heat

#### Five-trial heat box test

**Fig 3:**
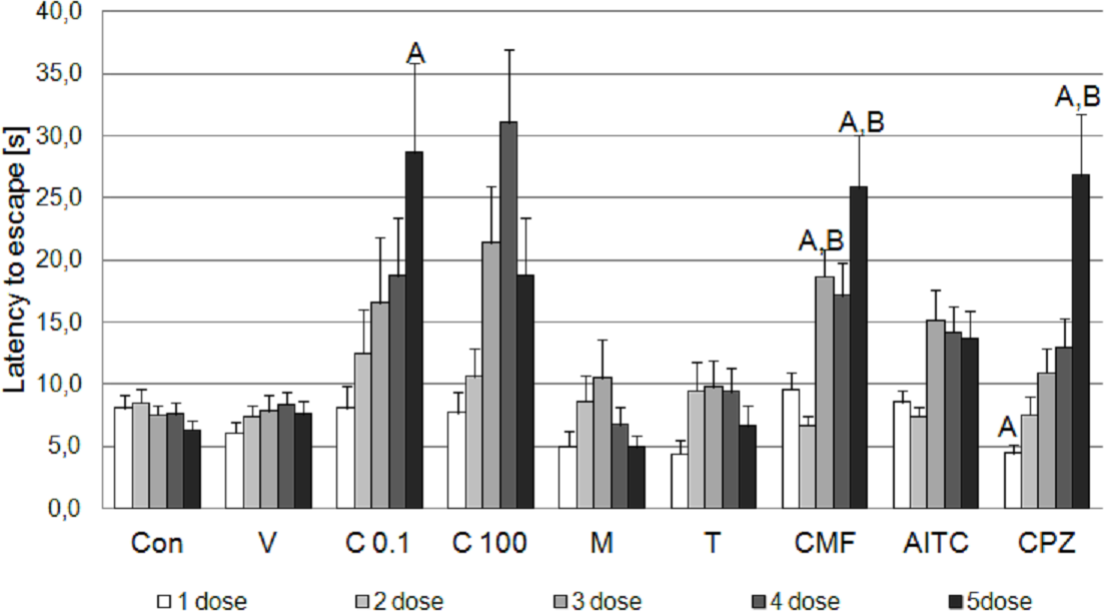
Five-trial heat box test. Latency to escape from noxious heat—50°C (s; mean ± SEM) after administration of water (Con), vehicle (V), capsaicin 0.0001mM (C 0.1) and 0.1mM (C100), menthol 2mM (M), thymol 1mM (T), camphor 15mM (CMF), allyl isothiocyanate 3mM (AITC) and capsazepine 0.0001mM (CPZ) in American cockroaches. Insects were exposed to the test compound and placed at 50°C once a day for five days–each dose administration and exposure to heat was repeated every 24 hours—five trial heat box test. Letters indicate results statistically significant vs. control (A) or solvent (B) group in each tested day (Mann-Whitney U test with Holm adjustment).

Cockroaches remained at 50°C for a significantly longer period of time after five doses of the test compound (Kruskal-Wallis test: χ^2^ = 40.8; df = 8; P<0.001) (Fig 3). Gradual prolongation of time spent at noxious heat, with each subsequent trial, was observed for cockroaches treated with capsazepine (26.8±4.9 s after the fifth dose; Mann-Whitney U test: U = 339, Z = -2.99, P = 0.003 vs. vehicle group) (Table 1). The latency to escape in cockroaches exposed to camphor was the longest after the fifth exposure as well (25.8±3.7 s; Mann-Whitney U test: U = 259, Z = -3.1, P = 0.002 vs. vehicle group), but the increase in time spent at noxious temperature was not linear. Significant differences were observed between the first and the third (Wilcoxon test: Z = -3.05, P = 0.002), the first and the fifth (Wilcoxon test: Z = -3.04, P = 0.002), the second and the third (Wilcoxon test: Z = -3.89, P<0.001), the second and the fourth (Wilcoxon test: Z = -3.27, P = 0.001) and between the second and the fifth dose (Wilcoxon test: Z = -3.18, P = 0.001) (Table 2). A small decrease between the first and the second dose and between the third and the fourth dose was observed, but the value of the latency to escape was effectively the same between these groups (non-significant changes).

**Table 1:** Results of statistical analysis of five-trial heat box test data.

			**The first dose**^1^^*^							
	n	Con 1x	V 1x	C 0.1 1x	C100 1x	M 1x	T 1x	CMF 1x	AITC 1x	CPZ 1x
Con 1x	60	-	0.38	0.22	0.14	0.13	0.02	0.53	0.40	**0.005**
V 1x	40	0.38	-	0.75	0.58	0.46	0.09	0.11	0.07	0.08
C0.1 1x	50	0.22	0.75	-	0.80	0.67	0.13	0.03	0.01	0.07
C100 1x	50	0.14	0.58	0.80	-	0.75	0.18	0.04	0.01	0.21
M 1x	19	0.13	0.46	0.67	0.75	-	0.50	0.04	0.02	0.63
T 1x	18	0.02	0.09	0.13	0.18	0.50	-	0.01	0.01	0.55
CMF 1x	60	0.53	0.11	0.01	0.04	0.04	0.01	-	0.69	**0.001**
AITC 1x	62	0.40	0.07	0.03	0.01	0.02	0.01	0.69	-	**0.001**
CPZ 1x	50	**0.005**	0.08	0.07	0.21	0.63	0.55	**0.001**	**0.001**	-
			**The third dose**^**1**^							
	n	Con 3x	V 3x	C 0.1 3x	C100 3x	M 3x	T 3x	CMF 3x	AITC 3x	CPZ 3x
Con 3x	55	-	0.68	0.36	0.07	0.62	0.18	**0.0003**	0.19	0.83
V 3x	35	0.68	-	0.15	0.05	0.38	0.26	**0.0003**	0.17	0.56
C0.1 3x	46	0.36	0.15	-	0.45	0.69	0.76	0.02	0.91	0.40
C100 3x	46	0.07	0.05	0.45	-	0.39	0.72	0.19	0.31	0.11
M 3x	16	0.62	0.38	0.69	0.39	-	0.75	0.03	0.74	0.80
T 3x	16	0.18	0.26	0.76	0.72	0.75	-	0.11	0.94	0.39
CMF 3x	50	**0.0003**	**0.0003**	0.02	0.19	0.03	0.11	-	0.09	**0.002**
AITC 3x	52	0.19	0.17	0.91	0.31	0.74	0.94	0.09	-	0.26
CPZ 3x	49	0.83	0.56	0.40	0.11	0.80	0.39	**0.002**	0.26	-
			**The fifth dose**^**1**^							
	n	Con 5x	V 5x	C 0.1 5x	C100 5x	M 5x	T 5x	CMF 5x	AITC 5x	CPZ 5x
Con 5x	46	-	0.46	**0.001**	0.02	0.35	0.86	**0.00007**	0.04	**0.00001**
V 5x	28	0.46	-	0.02	0.21	0.19	0.40	**0.002**	0.40	**0.003**
C0.1 5x	33	**0.001**	0.02	-	0.28	0.01	0.04	0.49	0.10	0.56
C100 5x	40	0.02	0.21	0.28	-	0.03	0.12	0.05	0.53	0.06
M 5x	13	0.35	0.19	0.01	0.03	-	0.50	**0.001**	0.08	**0.002**
T 5x	14	0.86	0.40	0.04	0.12	0.50	-	**0.003**	0.19	0.007
CMF 5x	34	**0.00007**	**0.002**	0.49	0.05	**0.001**	**0.003**	-	0.01	0.8
AITC 5x	45	0.04	0.40	0.10	0.53	0.08	0.19	0.01	-	0.01
CPZ 5x	42	**0.00001**	**0.003**	0.56	0.06	**0.002**	0.007	0.75	0.01	-

P-values for Mann-Whitney U test of data representing the latency to escape from 50°C in American cockroaches exposed to the first, third and fifth dose* of water (Con), vehicle (V), capsaicin 0.0001mM (C 0.1) and 0.1mM (C100), menthol 2mM (M), thymol 1mM (T), camphor 15mM (CMF), allyl isothiocyanate 3mM (AITC) and capsazepine 0.0001mM (CPZ). Values in bold show significant results after Holm adjustment (total number of tests to see whether drug-treated groups are comparable with respect e.g. vehicle group was N = 8).

^1^Kruskal-Wallis test results: the first dose: χ^2^ = 26.9, df = 8, p = 0.001; the second dose: χ^2^ = 8.52, df = 8, p = 0.39; the third dose: χ^2^ = 19.2, df = 8, p = 0.01; the fourth dose: χ^2^ = 15.0, df = 8, p = 0.06; the fifth dose: χ^2^ = 40.8, df = 8, p<0.0001.

* Mann-Whitney U test was not carried out for the second and fourth dose, as Kruskal-Wallis test revealed no significant differences between groups.

**Table 2:** Results of Wilcoxon-signed rank test for five-trial heat box test.

	First-second	First-third	First-fourth	First-fifth	Second-third	Second-fourth	Second-fifth	Third-fourth	Third-fifth	Fourth-fifth
Con	0.65	0.97	0.84	0.98	0.77	0.76	0.32	0.89	0.39	0.09
V	0.29	0.30	0.06	0.35	0.37	0.71	0.75	0.53	0.33	0.44
C 0.1	0.29	0.02	0.008	**0.0003**	0.37	0.08	0.01	0.35	**0.005**	0.27
C100	0.32	**0.001**	**0.0003**	0.03	0.04	0.04	0.18	0.37	0.69	0.73
M	0.04	0.07	0.3	0.86	0.96	0.68	0.15	0.16	0.29	0.20
T	0.04	0.06	0.16	0.33	0.84	0.73	0.3	0.68	0.20	0.43
CMF	0.12	**0.002**	0.04	**0.002**	**0.0001**	**0.001**	**0.001**	0.52	0.20	0.24
AITC	0.34	0.15	0.16	0.59	0.07	0.01	0.14	0.70	0.71	0.92
CPZ	0.14	**0.001**	0.01	**0.000003**	0.04	0.11	**0.0001**	0.70	**0.007**	0.03

P-values for statistical comparisons between the values obtained on different days (from first to fifth) within the same treatment, for each treatment with Wilcoxon signed-rank test. Values in bold show significant results after Holm adjustment (total number of tests to see whether groups treated on the one day treatment are comparable with groups treated on other tested days was N = 10). Cockroaches were treated with: water (Con), vehicle (V), capsaicin 0.0001mM (C 0.1) and 0.1mM (C100), menthol 2mM (M), thymol 1mM (T), camphor 15mM (CMF), allyl isothiocyanate 3mM (AITC) and capsazepine 0.0001mM (CPZ).

Differences in escape time between cockroaches treated with the fifth dose of capsaicin, camphor, and capsazepine were not significant, however camphor-induced latency to escape was significantly longer than in insects exposed to menthol and thymol. Capsazepine induced significant changes to menthol, but not thymol (Table 1). Cockroach responses to high ambient temperature were not significantly changed after multiple exposures to capsaicin, menthol, thymol, AITC, vehicle or water.

#### Single-trial heat box test

Capsaicin, camphor, and capsazepine induced changes in cockroach responses to high ambient temperatures, significantly prolonging time spent at 50°C (Kruskal-Wallis test: χ^2^ = 37.79; df = 8; P<0.001) (Table 3). The highest difference was observed for capsazepine (31.85 s longer than vehicle group; Mann-Whitney U test: U = 156.5, Z = -3.38, P = 0.001). However, there were no significant differences in time spent at noxious heat between cockroaches treated with capsazepine and capsaicin (Mann-Whitney U test: U = 210, Z = -1.69, P = 0.09 for 0.0001mM capsaicin and Mann-Whitney U test: U = 196, Z = -1.82, P = 0.07 for 0.1mM capsaicin) or camphor (Mann-Whitney U test: U = 272, Z = -1.63, P = 0.1). Menthol, thymol, and AITC did not influence cockroach escape time significantly.

**Table 3:** Single-trial heat box test.

Substance tested (five doses; one exposure to 50°C)	Con	V	C 0.1	C 100	M	T	CMF	AITC	CPZ
Latency to escape from heat box (s; mean±SEM)	3.54±0.49	4.36±0.54	17.86±4.28	14.51±2.99	5.23±1.17	7.17±1.89	18.34±3.98	7.62±1.83	36.21±8.64
Difference to control (water) group (s)	-	0.82	14.32*	10.97*	1.69	3.63	14.79*	4.08	32.67*
Difference to alcohol (solvent) group (s)	0.82	-	13.50*	10.14*	0.87	2.81	13.97^**^	3.25	31.85*
**Number of examined individuals**	35	37	31	30	16	18	39	39	19
Number of individuals that did not leave heat box	0	0	0	0	1	0	0	1	0
Number of dead individuals	1	1	3	1	3	0	1	0	0

Escape from heat box after administration with five doses (each dose applied every 24 hours) of water (Con), alcohol (V), capsaicin 0.0001mM (C 0.1) and 0.1mM (C100), menthol 2mM (M), thymol 1mM (T), camphor 15mM (CMF), allyl isothiocyanate 3mM (AITC) and capsazepine 0.0001mM (CPZ) in American cockroach. Insects were placed at 50°C only after the fifth dose of the test drug–single trial heat box test. Values are mean±SEM.

*indicates differences statistically significant to control and vehicle (bold) groups after Holm adjustment (total number of tests to see whether drug-treated groups are comparable with respect e.g. vehicle group was N = 8).

There is no significant difference in latency to escape between two respective groups–cockroaches exposed to single or five trial heat box test after the administration of the last fifth doses of the substances, except for vehicle-treated groups (Mann-Whitney U test: U = 318, Z = -2.65, P = 0.001).

### Cockroach behavioral thermoregulation after five doses of test compounds

**Fig 4:**
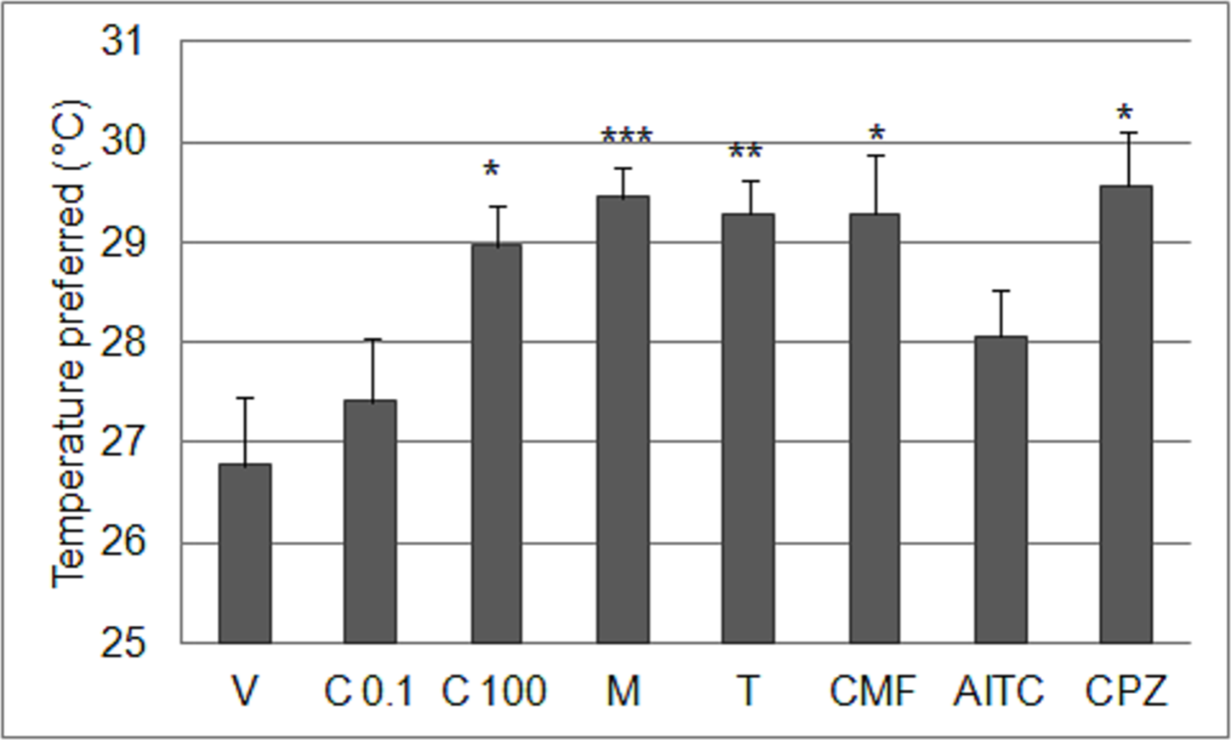
Behavioral thermoregulation in cockroaches after repeated compound administration. Ambient temperature preferred (°C; mean±SEM) by American cockroaches exposed to vehicle, capsaicin 0.0001mM (C 0.1) and 0.1mM (C100), capsazepine 0.0001mM (CPZ), menthol 2mM (M), thymol 1mM (T), camphor 15mM (CMF) and allyl isothiocyanate 3mM (AITC). Cockroaches were exposed to the test compounds for five days–each dose was repeated every 24 hours and then the insect was placed in the thermal gradient for 24 hours (n = 12 for each substance). * indicates values statistically significant from vehicle group (Mann-Whitney U test with Holm adjustment, * p<0.05; **p<0.01; *** p<0.001).

Cockroaches exposed to five dosings of a test compound (each drug was applied once every 24 hours) were able to freely choose ambient temperature in a thermal gradient. Vehicle-treated cockroaches spent most of time at a mean temperature of 26.77±0.15°C (Fig 4). Repeated administration of test drugs significantly impaired behavioral thermoregulation of cockroaches in the normal temperature range (Kruskal-Wallis test: χ^2^ = 36.84; df = 7; P<0.001). Drug-treated cockroaches preferred higher temperatures compared to vehicle-treated cockroaches after repeated-dosing with 0.1mM capsaicin (28.96±0.07°C; Mann-Whitney U test: U = 13, Z = -3.41, P = 0.001), menthol (29.45±0.1°C; Mann-Whitney U test: U = 1, Z = -4.1, P<0.001), thymol (29.28±0.11°C; Mann-Whitney U test: U = 8, Z = -3.7, P<0.001), camphor (29.28±0.15°C; Mann-Whitney U test: U = 11, Z = -3.52, P<0.001) or capsazepine (29.56±0.07°C; Mann-Whitney U test: U = 10, Z = -3.58, P<0.001).

### Effect of the test compounds on lipid peroxidation

As shown in Table 4, levels of lipid peroxidation were elevated in cockroaches exposed to a single-trial heat box test (Kruskal-Wallis test: χ^2^ = 45.84; df = 8; P<0.001). Significant increase in MDA levels were observed for capsaicin (0.1μM—Mann-Whitney U test: U = 29.0, Z = -3.85, P<0.0001 and 100 μM—Mann-Whitney U test: U = 17.0, Z = -4.47, P<0.001), menthol (Mann-Whitney U test: U = 16.0, Z = -4.42, P<0.001) and thymol-treated groups (Mann-Whitney U test: U = 30.0, Z = -3.82, P<0.001).

**Table 4:** Levels of oxidative stress marker (MDA) and catalase activity in cockroaches after five-trial and single-trial heat box tests.

	Con	V	C 0.1	C 100	M	T	CMF	AITC	CPZ
MDA (μmol/mg protein)									
**single-trial** heat box test	4.04	2.62	10.81	9.05	13.69	8.66	9.19	7.86	4.26
	±0.89	±0.63	±2.58	±0.84	±3.52	±1.66	±3.03	±1.78	±0.43
	N = 17	N = 16	N = 17	N = 19	N = 17	N = 17	N = 19	N = 20	N = 12
**five-trial** heat box test	13.94	10.91	10.82	7.91	7.45	2.60	9.63	6.52	6.87
	±3.16	±0.85	±1.43	±1.01	±1.52	±0.70	±1.46	±1.60	±1.60
	N = 21	N = 12	N = 12	N = 12	N = 8	N = 10	N = 11	N = 19	N = 12
Catalase activity (U/mg protein)									
**single-trial** heat box test	20.00	45.69	48.73	52.44	52.74	57.30	59.96	22.61	52.71
	±4.50	±7.75	±3.89	±2.51	±5.15	±2.68	±3.43	±3.03	±2.12
	N = 18	N = 17	N = 17	N = 19	N = 17	N = 17	N = 18	N = 20	N = 12
**five-trial** heat box test	40.48	88.49	105.9	65.60	63.38	62.69	87.46	17.9	81.73
	±2.02	±1.94	±1.17	±1.25	±1.20	±1.05	±1.46	±0.79	±1.55
	N = 15	N = 12	N = 12	N = 12	N = 12	N = 10	N = 11	N = 19	N = 12

Malondialdehyde concentration (MDA; μmol/mg protein) and catalase activity (U/mg protein) in cockroaches exposed to single-trial and five-trial heat box test treated with water (Con), alcohol (V), capsaicin 0.0001mM (C 0.1) and 0.1mM (C100), menthol 2mM (M), thymol 1mM (T), camphor 15mM (CMF), allyl isothiocyanate 3mM (AITC) and capsazepine 0.0001mM (CPZ). Values are mean±SEM. Underlined values are significant vs. vehicle group; Mann-Whitney U test after Holm adjustment; total number of test N = 8.

In the five-trial test cockroaches showed increased level of MDA comparing to single-trial test, but only in the control (U = 70, Z = -3.2, P = 0.001) and vehicle-alone (U = 6, Z = -4.2, P<0.001) groups, while thymol treatment resulted in MDA level decline after five doses (U = 13, Z = -3.6, P<0.001). Capsaicin, capsazepine, menthol and camphor did not affect MDA levels compared to the vehicle-treated group, however there was a significant decline in the lipid peroxidation marker level in thymol (U = 2, Z = -3.8, P<0.001) and AITC-treated groups (U = 29, Z = -3.4, P = 0.001).

### Effect of the test compounds on catalase activity

Cockroaches in the single-trial heat box test showed a significant increase in catalase activity versus the water-treated group (Kruskal-Wallis test: χ^2^ = 61.98; df = 8; P<0.001). However, there were no differences between vehicle (ethyl alcohol) and treated groups (Table 4). Similar results were obtained for cockroaches that underwent the five-trial heat box test (a non-significant difference between vehicle and treated groups, excluding the AITC).

Catalase activity in cockroaches exposed to five-trial test was significantly higher than that observed in insects exposed to single-trial heat box test in three drug groups (control: U = 39, Z = -3.1, P = 0.01; capsaicin 0.0001mM: U = 2, Z = -4.4, P<0.001; capsazepine: U = 6, Z = -3.78, P<0.001).

## Discussion

### Cockroach responses to noxious heat are affected by repeated doses of TRP ligands

Ambient temperature perception plays an essential role in the life of ectotherms, because it affects their ability to survive, reproduce, and develop. Perception of ambient temperatures allows insects to regulate their body temperature behaviorally, and therefore enables them to defend against variable temperatures. Our experiments showed that some TRP agonists and antagonists: capsaicin, capsazepine, and camphor may induce changes in insect perception and response to high ambient temperatures.

Pharmacological blockade of heat sensitivity, observed as a weaker response to noxious heat, occurred in cockroaches exposed to five doses of capsazepine. Although capsazepine is one of the most extensively studied TRPV1 antagonists, it is characterized by moderate potency and limited selectivity [42]. Jakab et al. [42] demonstrated that while 1 and 10μM capsazepine causes inhibition of capsaicin-activated Ca^2+^ influx in trigeminal ganglion neurons, 30μM capsazepine acts as a partial agonist to capsaicin action in these cells. Kistner et al. [24] showed that capsazepine induced desensitization in mice through activation of TRPA1 receptors. Moreover, there are reports showing that capsazepine is not only a TRPV1 antagonist, but also inhibits the TRPM8 response to menthol [43]. Our results suggest that in cockroach capsazepine may act on structures other than TRPV, presumably some members of TRPA, but further electrophysiological studies are needed to clarify this hypothesis.

Our results demonstrate that cockroaches exposed to repeated doses of camphor tend to stay much longer at high ambient temperature (50°C) than insects that were not exposed to the alkaloid. This suggests that cockroaches treated with camphor became, to some extent, insensitive to noxious ambient temperatures. Contrary, it can be considered that the sensitized state to the action of this compound occurred in the examined insects, as the absence of the effect on escape latency after first administration was observed, and the development of the substantial effect on escape latency after the fifth administration. Camphor is a product of the fragrant camphor tree and is known for its antimicrobial and anti-nociceptive effects [44]. It is suggested that analgesic effects of camphor are mediated by desensitization of TRPV1 and inhibition of TRPA1 [9]. It was also demonstrated that in insects, camphor can activate cold receptor TRPL and inhibit Painless, which responds to noxious heat [29]. Camphor was also reported to activate Hymenoptera-specific TRPA (Hymenoptera equivalent of dTRPA1), and is suggested to activate *Drosophila* warmth receptor dTRPA1 [26].

Capsaicin also changed cockroaches’ response to high ambient temperature, however the significant effect was observed only in single-trial heat box test. Capsaicin is known to activate the mammalian heat receptor TRPV1, and in high doses it causes TRPV1 desensitization and loss of the ability to perceive high ambient temperatures [1]. However, there is limited data concerning the effect of capsaicin on insect thermo-TRP. In vitro results showed that insect vanilloid receptors (Nanchung and Inactive) do not respond to capsaicin [45]. On the other hand, capsaicin is often used as an insect repellent [46]. Experiments conducted on mealworm larvae and on the American cockroach using a thermal gradient showed that insects respond to capsaicin and capsazepine by modifying their thermal preferences [36;47]. Mealworms intoxicated with capsaicin or capsazepine responded similarly to mammals (in mealworms, capsaicin induced preference for lower temperatures, while capsazepine for higher temperatures). Similar findings were recently published by Zermoglio et al. [48] and showed that *Rhodnius prolixus* treated with capsaicin is less responsive to heat stimuli, fails to orient in space, and prefers lower temperatures than non-treated insects, while capsazepine induced opposite behaviors.

To ascertain that the diminished heat sensitivity in capsaicin, capsazepine and camphor-treated cockroaches is not an effect of other possible causes, such as general toxicity, central nervous suppression or partial motor paralysis, we conducted additional behavioral test–response of exposed insects to noxious cold. Data are presented inS5 Spreadsheet. Latency to escape from 5°C in treated cockroaches was measured and the results demonstrated that capsaicin, capsazepine and camphor do not affect cockroaches response to noxious cold comparing to the vehicle group.

AITC is known to activate two warmth receptors in insects–dTRPA1 and Painless, in vitro [26;30]. However, the effects of AITC on Painless are contradictory. Wild-type *Drosophila* tended to avoid food containing AITC, while *painless* mutants did not show such avoidance behavior. HEK293 cells expressing Painless were unresponsive to AITC, temperature (10°C), capsaicin, menthol, or camphor. It is possible that interaction with accessory proteins, formation of heterometric channel, or splice variants may modify this channel’s properties in vivo [29]. TRP channels may interact with accessory proteins to form complexes, and the components of these complexes probably regulate gating and localization of TRP within specialized membrane domains [49]. It was demonstrated that *Helicoverpa armigera* TRPA1 (HarmTRPA1) is activated in vitro by AITC and also functions as a warmth sensor [31]. There are reports showing that Painless becomes desensitized when repeated heat stimuli are applied [29]. AITC was demonstrated to induce a progressive increase in withdrawal latencies to radiant heat stimulation during three to five days of administration, which normalized after two weeks of wash-out in mice [24]. It was also shown that local TRPA1 desensitization by AITC suppressed the inflammatory reaction in an allergic sensitivity mouse model [50]. AITC was also shown to directly activate TRPV1 and induce hypersensitivity to heat in mice [51]. However, our results did not show any significant changes in cockroach responses to noxious heat after AITC exposure. Menthol and thymol, which are known to activate the insect cold receptor TRPL [25], also did not affect cockroach responses to high ambient temperature.

It is worth emphasizing that cockroaches, after exposure to five doses of capsazepine and camphor, showed a significant increase in time spent at 50°C, irrespectively of type of experimental schedule–single or five-trial heat box test. This implies that these drugs may facilitate development of a strong suppression of the heat sensitivity. The effect of capsaicin on escape latency was less pronounced. This may suggest development of the sensitized state to the action of these compounds or on the other hand, a desensitized state to noxious heat. Babcock et al. [52] reported that thermal hyperalgesia, observed as heightened sensitivity to noxious heat, occurred in *Drosophila* when 95% of larvae escaped from 45°C in less than 5 seconds comparing to 26% in control group. In our study the increase in latency to escape from noxious heat was observed, which may suggest thermal hypoalgesia induced by alkaloids treatment.

### Behavioral thermoregulation of ‘sensitized’ cockroaches in normal temperature ranges is altered

Cockroaches exposed to repeated dosing of the drugs showed a preference for higher ambient temperatures versus the vehicle-treated group and control (water-treated) group. The interesting observation was that although submicromolar capsaicin induced changes in cockroach thermoregulation against noxious heat in single-trial heat box test, it did not significantly affect thermal preferences in normal temperature range. Cockroaches treated with five doses of submicromolar capsaicin prefer similar temperatures to non-treated insects (Fig 4), but after one treatment of the same dose of this alkaloid a hypothermic effect was observed [47]. The results show that the effect of capsaicin on cockroach thermoregulation is dose-dependent (one dose–hypothermic effect, five doses–no effect on thermal preferences). It seems that, after multiple capsaicin exposures, cockroaches become less sensitive not only to high ambient temperatures, but also to the hypothermic effect of capsaicin action. It was shown that capsaicin-desensitized rats, apart from impaired tolerance to high ambient temperature, demonstrated decreased sensitivity towards the hypothermic effect of this alkaloid [53]. The results obtained here show that the thermal response of capsaicin-exposed cockroaches differs dependently on temperature. In the normal temperature range (10–40°C) capsaicin-‘sensitized’ cockroach behavioral thermoregulation is intact, while at high ambient temperature (50°C) it may become altered. This is consistent with results observed in mammals, in which capsaicin desensitization did not induce consistent changes in body or skin temperature. Other studies also showed that the effects of capsaicin desensitization on body temperature were typically small, relatively short-lived, and difficult to find, whereas the effects on the responses to heat were profound and reproducible [13].

Therefore, the question arises, why do cockroaches exposed to multiple doses of 0.1 mM capsaicin, capsazepine, and camphor tend to stay in warmer regions of a thermal gradient, since they also demonstrate signs of suppression of the heat sensitivity to noxious heat? It may be assumed that capsazepine and camphor may act on structures other than TRPV. Capsazepine was shown to activate TRPA1 [24]. Non-specific action of capsazepine was demonstrated in other studies. Docherty et al. [54] demonstrated that capsazepine at 1.4μM concentration was able to block voltage-activated calcium channels. However, capsazepine is 10-fold more potent as a capsaicin antagonist than as a calcium current blocker. Nicotinic acetylcholine receptors in rat trigeminal ganglia were also blocked by 10μM capsazepine [55]. Capsazepine (EC_50_ value = 8μM) is also the first known chemical activator of human amiloride-sensitive epithelial Na^+^ channels [56]. It also blocks potassium channels in embrio spinal neurons of *Xenopus* [57].

It seems that the effect of capsazepine action is also highly species-specific. It was demonstrated that human and guinea pig TRPV1 response to capsaicin, noxious heat, and protons was inhibited by capsazepine, while for rat TRPV1 capsazepine blocked the response only to capsaicin, but not to low pH (the blockade of response to heat was weaker) [58]. Capsazepine was not reported to cause hyperthermia in the rat or mouse, although TRPV1 antagonists were shown to induce hyperthermia in mammals. The hyperthermic effect occurs when an antagonist inhibits capsaicin and proton activation. Because capsazepine does not block activation of TRPV1 by protons in the rat or mouse, it does not induce hyperthermia, however it induced this effect in guinea pigs, where it blocked TRPV1 proton-induced activation [13]. Camphor inhibits Painless receptor in Drosophila [29]. Inhibition of a receptor responsible for detection of noxious heat would explain the cockroach responses observed in this study. However, the structures in cockroaches that are affected by camphor are unknown. Multiple administrations of menthol and thymol induced cockroach preference for warmer regions, while their thermoregulation in noxious heat was not altered. It was shown that these drugs, in vitro, inhibit insect cold receptors [25]. In bees, menthol blocks warm receptor HsTRPA and induces preference for warmth [26]. However, these results were obtained for a single dose of the drugs. Repeated administration may induce distinct mechanisms and physiological responses.

### Oxidative stress is not responsible for impaired heat nociception

TRPV1 activation by capsaicin was demonstrated to increase subsequent oxidative stress, which may contribute to elevated pain sensation [32]. Reactive oxygen species mediate the development of capsaicin-induced hyperalgesia in mice [33], although capsaicin was shown to exert also some antioxidant effect [59]. Suppression of oxidative damages were also reported for menthol, thymol and AITC [60–62].

In order to determine whether the cockroach response to noxious heat could be affected by a secondary effect of the drugs, we examined the level of lipid peroxidation and catalase activity as markers of oxidative state. In the single-trial heat box test cockroaches treated with capsaicin showed an increase in MDA levels, suggesting that this drug exert some toxic effect. However, higher MDA levels were also observed in cockroaches exposed to menthol or thymol, neither of which affected cockroach response to heat. Moreover, in cockroaches exposed to heat for five trials, some drugs (thymol and AITC) significantly lowered the MDA level, irrespectively of action on thermal perception. Therefore, we can exclude that the increased oxidative stress induced significant changes in cockroach heat response. We could also mention that thymol and AITC were shown to exhibit some antioxidant activity [61–62] and capsazepine revealed neuroprotective action against oxidative stress in cultured hippocampal neurons [63].

Our results demonstrate that an increase in time spent at 50°C (single versus five heat box trials) induces oxidative stress, but only in control and vehicle- treated groups. The levels of MDA in capsaicin, capsazepine, menthol, camphor and AITC-treated cockroaches did not differ between one or multiple exposures to heat. Moreover, the tested drugs did not affect catalase activity compared to vehicle, irrespectively of time spent at noxious heat (a single trial or five trials). Only AITC induced a significant reduction in catalase activity in cockroaches exposed to 50°C during five trial test. AITC was shown to reduce oxidative stress and induce increase in glutathione-S-transferase activity in *C*. *elegans* [62].

The toxicity of the tested compounds was low. The observed mortality levels of cockroaches exposed to five doses of the test drugs was appreciable, but appears to have resulted not from the drugs but from vehicle and the experimental paradigm itself, as the highest 15% mortality occurred after vehicle-alone and camphor treatments only. Capsazepine, which induced the strongest sensitization effect, induced only 8% mortality. Other studies confirm low fumigant and contact toxicity of camphor in German cockroach [64] and red flour beetle [65].

## Conclusions

Our results show, for the first time, that repeated exposure to capsazepine, camphor and capsaicin induces a state of pharmacological blockade of heat sensitivity. Cockroaches became sensitized to the action of these compounds what is observed as the development of the substantial effect on escape latency after the fifth administration. Moreover, behavioral thermoregulation of such ‘sensitized’ insect in normal temperature range is altered.

## Supporting information

S1 Spreadsheet Excel spreadsheet showing results from heat box experiment. Latency to escape from noxious heat—50°C (s; raw data) after administration of water (Con), vehicle (V), capsaicin 0.0001mM (C 0.1) and 0.1mM (C100), menthol 2mM (M), thymol 1mM (T), camphor 15mM (CMF), allyl isothiocyanate 3mM (AITC) and capsazepine 0.0001mM (CPZ) in American cockroaches. Single and five-trial heat box test.
(XLSX)

S2 Spreadsheet Excel spreadsheet showing results from thermal gradient experiment. Temperatures preferred by cockroaches exposed to test drugs after repeated application (5 doses).
(XLSX)

S3 Spreadsheet Malondialdehyde levels in cockroaches. Malondialdehyde concentration (MDA; μmol/mg protein) in cockroaches exposed to single-trial and five-trial heat box test treated with water, alcohol, capsaicin 0.0001mM and 0.1mM, menthol 2mM, thymol 1mM, camphor 15mM, allyl isothiocyanate 3mM and capsazepine 0.0001mM.
(XLSX)

S4 Spreadsheet Catalase activity in cockroaches. Catalase activity (U/mg protein) in cockroaches exposed to single-trial and five-trial heat box test treated with water, alcohol, capsaicin 0.0001mM and 0.1mM, menthol 2mM, thymol 1mM, camphor 15mM, allyl isothiocyanate 3mM and capsazepine 0.0001mM.
(XLSX)

S5 Spreadsheet Cold nociception assay. Latency to escape from noxious cold—5°C (s; raw data) after administration of water (Con), vehicle (V), capsaicin 0.0001mM (C 0.1) and 0.1mM (C100), menthol 2mM (M), thymol 1mM (T), camphor 15mM (CMF), allyl isothiocyanate 3mM (AITC) and capsazepine 0.0001mM (CPZ) in American cockroaches. Single-trial cold plate test.
(XLSX)
